# Structure and Genome Organization of a Novel Fiji Strain of Sweet Potato Vein Clearing Virus Identified by High-Throughput Sequencing

**DOI:** 10.1128/genomeA.00462-18

**Published:** 2018-06-14

**Authors:** Liping Wu, Huawei Liu, Jorge Abad, Ronald D. French, Ruhui Li

**Affiliations:** a USDA-ARS, National Germplasm Resources Laboratory, Beltsville, Maryland, USA; b Key Laboratory of Poyang Lake Environment and Resource, Nanchang University, Jiangxi, China; c USDA-APHIS, Plant Protection and Quarantine, Riverdale, Maryland, USA

## Abstract

The complete genome of a Sweet potato vein clearing virus (SPVCV) isolate infecting a quarantined sweet potato accession from Fiji was determined. Sequence comparisons revealed the highest nucleotide sequence identity of 94.6% with that of the SPVCV type species, an isolate from the Dominican Republic. The virus was mechanically transmitted to Nicotiana bigelovii plants.

## GENOME ANNOUNCEMENT

Sweet potato vein clearing virus (SPVCV), a member of genus Solendovirus in the family Caulimoviridae, has been reported from Africa and the Americas (1). Its icosahedral virions (50 nm in diameter) contain an open circular, double-stranded DNA of 8.8 kb. Although the virus does not induce obvious symptoms on sweet potato (Ipomoea batata), mixed infections with other viruses cause disease. While undergoing quarantine testing in 2016, a sweet potato accession imported from Fiji displayed chlorotic and necrotic leaf spots. When the accession was grafted onto indicator I. setosa, vein clearing and necrosis were observed. To identify the causal agent, total RNA was isolated from the accession plant with the RNeasy plant minikit (Qiagen, USA) and subjected to Illumina NextSeq 500 sequencing (SeqMatic, USA). Plant rRNA was removed using the Illumina Ribo-Zero rRNA removal kit before cDNA library construction. Analysis of total RNA reads was performed using CLC Genomics Workbench version 9.5.2. Contigs were annotated by BLASTx comparisons. *De novo* assembly of the RNA reads generated 25,070 contigs (>200 nucleotides [nt]) from the sample, and a BLASTx search of the contigs against the Viruses_NR database revealed the presence of a large contig (8,746 nt) with high amino acid (aa) sequence identities (93 to 98%) to Sweet potato vein clearing virus (SPVCV) (GenBank accession number NC_015228). A total of 64,960,069 reads (7,427× coverage, 2.3% total reads) mapped to this contig, supporting the infection of SPVCV in the accession. No other virus, viroid, or phytoplasma was identified. The virus genome (GenBank accession number MH188860) was resequenced using plasmid DNA cloned from PCR products that were amplified using virus-specific primers. The genome of this Fijian isolate shared 94.6% identity with Dom1, a SPVCV isolate from the Dominican Republic.

The genome of the Fijian SPVCV isolate (8,746 nt) is 87 nt shorter than that of Dom1, mainly due to several deletions around the initial replication site, but its structure is virtually identical to that of Dom1. The aa sequence identities between the two isolates are 90.5% at the coat protein, 96.9% at the movement protein, 98.7% at the replicase polyprotein, and 94.8% at the inclusion body protein. The virus was mechanically transmitted to Nicotiana bigelovii plants, as confirmed by PCR using virus-specific primers. This is the first report and determination of a complete genome of a SPVCV isolate from the South Pacific islands.

### Accession number(s).

The complete genomic sequence of the Fijian isolate of SPVCV has been deposited in GenBank under the accession number MH188860.
